# The Limited Role of Hypocortisolism in the Development of Delayed Postoperative Hyponatremia After Pituitary Surgery

**DOI:** 10.1007/s11102-026-01702-9

**Published:** 2026-06-16

**Authors:** Jong Ha Hwang, Hye Seok Park, Sun Mo Nam, Seung Shin Park, Jung Hee Kim, Min-Sung Kim, Chul-kee Park, Yong Hwy Kim

**Affiliations:** 1 Department of Neurosurgery, Seoul National University Hospital, Seoul National University College of Medicine, 101 Daehak-ro, Jongno-gu, Seoul, 03080 Republic of Korea; 2 Division of Endocrinology and Metabolism, Department of Internal Medicine, Seoul National University Hospital, Seoul National University College of Medicine, Seoul, 03080 Republic of Korea

**Keywords:** Hyponatremia, Endoscopic endonasal approach, Adrenal insufficiency, Pituitary neoplasms

## Abstract

**Purpose:**

Delayed postoperative hyponatremia (DPH) is a common complication following pituitary surgery. Although hypocortisolism has been proposed as a contributing factor, the temporal interplay between cortisol and sodium levels remains unclear. This study aimed to elucidate the relationship between postoperative cortisol and sodium dynamics, identify independent predictors of DPH and assess potential protective factors.

**Methods:**

We retrospectively analyzed 261 patients who underwent fully endoscopic surgery for clinically non-functioning pituitary neuroendocrine tumors (Pit-NETs, also known as pituitary adenomas) between 2021 and 2024. Serial measurements of serum sodium and morning cortisol were collected from the immediate postoperative period through 3-month follow-up. Comparative analyses between patients with and without DPH were performed to characterize hormonal and electrolyte trends. The frequency of hypocortisolism during hyponatremic episodes was compared, and multivariable logistic regression identified independent predictors. The potential protective role of preoperative steroid replacement was also evaluated.

**Results:**

DPH occurred in 54 patients (20.7%), with symptomatic cases accounting for 4.2% of the cohort. The median time to nadir sodium was 8 days with a median nadir of 129 mEq/L. The serum cortisol level between hyponatremia and non-hyponatremia group did not show significant differences. For propensity score matched comparison, median nadir cortisol levels were lower in the DPH group (6.10 vs. 7.34 µg/dL, *p* = 0.29), and hypocortisolism during hyponatremic episodes was more frequent (48.2% vs. 40.7%, *p* = 0.52), though neither difference reached statistical significance. Multivariate analysis identified age > 60 years (OR 2.09, 95% CI 1.11–3.96, *p* = 0.023) and postoperative pneumocephalus (OR 3.28, 95% CI 1.59–6.81, *p* = 0.001) as independent predictors. Preoperative cortisol replacement did not demonstrate a protective effect against DPH.

**Conclusion:**

Hypocortisolism does not appear to be the primary mechanism underlying DPH following pituitary surgery, as evidenced by the lack of temporal correlation. Advanced age and postoperative pneumocephalus emerge as key risk factors for DPH, supporting a multifactorial etiology. Although preoperative ACTH deficiency was linked to a higher incidence of DPH, preoperative cortisol replacement was not associated with protection.

**Supplementary Information:**

The online version contains supplementary material available at https://doi.org/10.1007/s11102-026-01702-9.

## Introduction

Delayed postoperative hyponatremia (DPH) is a common yet frequently underrecognized complication after pituitary tumor surgery, with reported incidence rates ranging from 4% to 28% [1–3]. Defined as hyponatremia occurring on or after postoperative day (POD) 3, it typically manifests with nonspecific symptoms such as lethargy, nausea, or headache, but severe cases may cause confusion, seizures, or loss of consciousness. The onset usually occurs between postoperative days 7 and 10, when most patients have been discharged, making DPH a major cause of readmission [4, 5]. Given its potential for rapid clinical deterioration, timely detection and thorough understanding of its underlying mechanisms are essential.

The pathophysiology underlying DPH is multifactorial and remains incompletely understood. Proposed mechanisms include syndrome of inappropriate antidiuretic hormone secretion (SIADH), cerebral salt wasting, primary polydipsia, hypothyroidism, and hypocortisolism [6]. Among these mechanisms, SIADH has been regarded as the principal driver, forming the basis for postoperative fluid restriction as a preventive strategy. This approach has demonstrated effectiveness and is now widely implemented [7, 8]. Nevertheless, despite perioperative fluid management, DPH continues to occur, underscoring the need to identify additional biological factors contributing to its development.

Hypocortisolism is one such factor, as cortisol deficiency promotes antidiuretic hormone (ADH) secretion and enhances renal sensitivity to ADH, thereby predisposing patients to hyponatremia [9, 10]. Although previous studies have suggested a potential association between postoperative cortisol dynamics and DPH, the relationship has not been examined through a comprehensive quantitative evaluation.

Accordingly, the primary aim of this study was to elucidate the relationship between postoperative serum cortisol and sodium levels following pituitary surgery. The secondary objectives were to identify reliable predictors of DPH and to assess the influence of preoperative ACTH deficiency and cortisol replacement therapy.

## Materials and methods

### Study population

This retrospective single-institution study was approved by the Institutional Review Board (IRB No. 2507-064-1656) and conducted in accordance with the Declaration of Helsinki. The requirement for informed consent was waived due to the retrospective design of the study.

Patients were drawn from a prospective pituitary adenoma observation cohort (IRB No. 1503-040-654) established in 2015. We included individuals with pathologically confirmed pituitary neuroendocrine tumors (Pit-NETs, also known as pituitary adenomas), who underwent fully endoscopic surgery between 2021 and 2024. The study focused on clinically non-functioning pituitary tumors. Patients with functioning tumors, including Cushing’s disease (*n* = 25), acromegaly (*n* = 46), and prolactinoma (*n* = 14), were excluded. To minimize confounding effects on postoperative sodium levels, patients with untreated hypothyroidism or preoperative diuretic use were excluded.

### Management protocol

At our institution, surgery is performed for clinically non-functioning pituitary adenomas/Pit-NETs when patients are symptomatic or at imminent risk of developing symptoms, most commonly due to visual field impairment from optic chiasm compression. No specific tumor size criteria were used for surgical decision-making, as the distance between the sellar floor and the optic nerve or chiasm varies among individuals, and tumors extending exclusively in an inferior direction do not produce clinically significant effects. Visual symptoms were assessed based on visual field testing, supported by retinal nerve fiber layer thinning on optical coherence tomography. The most common endocrinologic indications for surgery included hypocortisolism, hyperprolactinemia due to stalk compression effect in women of reproductive age, and sex hormone deficiency in male patients. Preoperative evaluation includes comprehensive endocrinologic and ophthalmologic assessments. The preoperative endocrinologic workup consisted of basal hormone studies, including morning serum cortisol, free T4, TSH, prolactin, LH, FSH, estradiol or testosterone, and IGF-1 [11–13]. Morning serum cortisol was measured at 6:00 AM to 8:00 AM. In patients receiving hydrocortisone replacement, the last oral dose was administered at 5:00 PM the previous day, ensuring a minimum interval of more than 12 h between the last dose and blood sampling. Given that the plasma half-life of cortisol is approximately 60–90 min, a 12-hour interval allows near-complete clearance of exogenous hydrocortisone, minimizing interference with cortisol measurements [14, 15].

A rapid adrenocorticotropic hormone (ACTH) stimulation test was performed to evaluate the hypothalamic-pituitary-adrenal (HPA) axis, except in patients with confirmed hypopituitarism. Even in patients with low basal cortisol levels, the stimulation test was performed when the basal measurement was not obtained under optimal conditions (fasting state, before 8:00 AM), as a single basal cortisol measurement may not accurately reflect adrenal reserve. In patients presenting with pituitary apoplexy or symptomatic hypocortisolism, hydrocortisone replacement was initiated immediately without prior stimulation testing. When basal screening suggested possible hormonal hypersecretion, confirmatory tests were selectively performed: UFC and dexamethasone suppression test for suspected Cushing’s disease, and oral glucose tolerance test for suspected acromegaly.

Perioperative steroid management is guided by preoperative rapid ACTH stimulation test results. Patients with preoperative ACTH deficiency received 100 mg of intravenous hydrocortisone one hour before surgery. Postoperatively, intravenous hydrocortisone 25 mg was administered every 6 h on the day of surgery. On postoperative day 1, hydrocortisone was transitioned to oral form (30 mg in the morning and 20 mg in the evening) and subsequently tapered to a physiologic dose (10 mg in the morning and 5 mg in the evening) at discharge. During admission, morning serum cortisol was measured before the morning hydrocortisone dose, and the replacement dose was adjusted if the cortisol level was below 8 µg/dL. Patients with a normal ACTH stimulation test result, defined as a peak cortisol level higher than 18 µg/dL [11, 16], and adequate postoperative cortisol levels do not receive perioperative corticosteroid supplementation. Postoperative imaging includes immediate CT and MRI within 48 h to assess acute complications and residual tumor. Standard discharge occurs on postoperative day 3, followed by outpatient assessments at weeks 1, 2, 4, and thereafter. Upon discharge, patients without arginine vasopressin (AVP) deficiency are instructed to restrict daily fluid intake to less than 1 L. In patients with diagnosed AVP deficiency, oral desmopressin is prescribed instead of fluid restriction. As postoperative AVP deficiency is mostly transient, patients are instructed to begin fluid restriction to less than 1 L per day once desmopressin is no longer required. Serum electrolytes are routinely monitored twice daily after surgery during admission and serially monitored at the outpatient clinic. When DPH is detected, management is individualized according to serum sodium levels and symptom severity; severe cases require readmission for controlled fluid and electrolyte correction, including administration of 3% saline when clinically indicated.

### Data collection and outcome measures

The primary objective was to evaluate the relationship between serum sodium and cortisol levels in the context of DPH. Sodium and cortisol measurements were analyzed across predefined postoperative intervals: day 0 (immediate postoperative period), days 1–3 (inpatient period), and outpatient follow-up periods categorized as early follow-up (1–3 weeks) and late follow-up (3 weeks to 3 months). All hormone measurements, including cortisol, were performed in the fasting state at approximately 8:00 a.m. to minimize diurnal variation. We modeled serum sodium and cortisol trends using generalized additive mixed models (GAMMs), allowing flexible nonlinear smoothing of temporal patterns and visualization of population-level changes.

For comparing nadir cortisol levels between patients with and without DPH, a time-window–matched analysis was conducted. To reduce baseline confounding, patients were matched 1:1 using propensity scores. For each patient who developed hyponatremia, the nadir cortisol level during the hyponatremic episode was compared with the cortisol level of their matched control from the identical postoperative time window. Hypocortisolism was defined as a nadir morning cortisol level < 5 µg/dL, given the limited reliability of the ACTH stimulation test in the immediate postoperative period [15, 17]. The proportion of hypocortisolism was subsequently compared between groups.

Risk factors for DPH were then evaluated using patient-specific and tumor-specific variables. Patient characteristics included age, sex, body mass index (BMI), comorbidities, and body composition parameters (total body water, fat, and skeletal muscle) assessed via bioimpedance analysis. Surgical factors included the postoperative pneumocephalus, operation time, and extent of resection. Tumor-related factors included preoperative hormonal deficiencies, presence of pituitary apoplexy, recurrence status, Knosp grade, tumor volume, and pituitary cell lineage classification according to the 2022 WHO classification (PIT-1, T-PIT, SF-1) [18]. Finally, the effects of preoperative ACTH deficiency and the potential protective influence of cortisol replacement therapy were assessed.

### Definitions

DPH was defined as serum sodium < 135 mEq/L starting on or after postoperative day 3. Resolved early hyponatremia followed by new onset after day 3 was also considered DPH. Severity was classified as mild (130–135 mEq/L), moderate (125–130 mEq/L), or severe (< 125 mEq/L) [19].

Clinically non-functioning tumors were defined by excluding clinical Cushing’s disease, prolactinoma, acromegaly, and thyroid-stimulating hormone (TSH)-secreting tumors to eliminate confounding effects on cortisol levels. Perioperative steroid replacement is defined as more than two doses of hydrocortisone before POD 3.

### Statistical analysis

Analyses were performed using Python (version 3.12.0). Continuous variables were assessed for normality using the Shapiro-Wilk test and compared using t-tests or Mann-Whitney U tests as appropriate. GAMM-based analysis was conducted using R (version 4.5.0) with the mgcv package, incorporating patient-specific random intercepts to account for repeated measurements. The propensity score, which represents the predicted probability of developing DPH, was calculated for each patient using a multivariate logistic regression model that included key baseline covariates. Patients were then matched using a nearest-neighbor algorithm on the logit of the propensity score without replacement. Adequate balance of all covariates after matching was confirmed by ensuring that all standardized mean differences (SMDs) were below the threshold of 0.1. Risk factor analysis employed logistic regression, with multivariable analysis including both statistically and clinically significant factors from univariable analysis. A complete-case analysis was performed, as the proportion of missing data was less than 5%.

## Results

### Patient demographics

Among 281 patients, 20 were excluded due to diuretic use. The final cohort of 261 patients had a median age of 57 years, with a nearly equal gender distribution (131 males, 130 females). Six patients presented with pituitary apoplexy, and 32 had recurrent tumors. Growth hormone deficiency was the most prevalent endocrine deficiency (60.9%), followed by gonadotropin (60.2%), ACTH (32.6%), and TSH deficiencies (10.3%) (Table 1).

**Table 1:** Baseline characteristics of the study cohort (*N* = 261)

Variable	Total
Demagraphics	
Age, years	57 [46–66]
Sex, male: female	131: 130
BMI, kg/m^2^	25.2 [23.0–27.8]
Comorbidities	
Hypertension	80 (30.7)
Diabetes Mellitus	31 (11.9)
Dyslipidemia	84 (32.2)
Cardiac disease	17 (6.5)
Pulmonary disease	8 (3.1)
Hepatic disease	4 (1.5)
Renal disease	7 (2.7)
Preoperative endocrinologic deficiencies	
ACTH deficiency	85 (32.6)
Gonadotropin deficiency	157 (60.2)
TSH deficiency	27 (10.3)
GH deficiency	159 (60.9)
Tumor-related factors	
Pituitary apoplexy	6 (2.3)
Recurrent tumor	32 (12.3)
Tumor volume, cm^3^	5.60 [3.43–9.30]
Knosp grade	
0	24 (9.2)
1	75 (28.7)
2	62 (23.8)
3a	60 (23.0)
3b	8 (3.1)
4	32 (12.3)

Values are expressed as number (%), or median [interquartile range] for continuous variables

*ACTH* adrenocorticotropic hormone, *BMI* body mass index, *GH* growth hormone, *TSH* thyroid-stimulating hormone

### Incidence, characteristics and management of DPH

DPH occurred in 54 patients (20.7%), with symptomatic cases accounting for 4.2% of the total cohort. Cases were classified by severity: mild (21/52, 40.4%), moderate (22/52, 40.4%), and severe (11/52, 19.2%). The proportion of symptomatic patients increased with severity of hyponatremia (0% in mild, 22.7% in moderate, and 54.5% in severe cases). Similarly, readmission rates showed a marked increase with severity (4.8%, 13.6%, and 36.4%, respectively). The median time to lowest serum sodium was 8 days, with a median nadir of 129 mEq/L (Online Resource 1). All cases except one occurred within a month after the operation. The symptoms included headache, nausea, poor oral intake, vomiting, dizziness, general weakness, somnolence, visual disturbance, and drowsy mentality. Two patients presented with drowsy mentality as the most severe manifestation.

Treatment approaches varied by severity of hyponatremia. Observation alone was limited to mild cases (4/21, 19%), while all moderate and severe cases required active intervention. Steroid replacement was the predominant intervention across all groups (76.2%, 95.5%, and 90.9% in mild, moderate, and severe cases, respectively). 3% saline administration increased markedly with severity: only one patient (4.8%) in the mild group required 3% saline, compared to 40.9% of moderate and 36.4% of severe cases. One patient with mild hyponatremia received hypertonic saline in the context of a complicated postoperative course due to meningitis. Notably, all symptomatic patients had nadir sodium levels below 129 mEq/L. No patients developed severe complications such as seizures or altered mental status.

### Serum sodium and cortisol dynamics

**Fig. 1:**
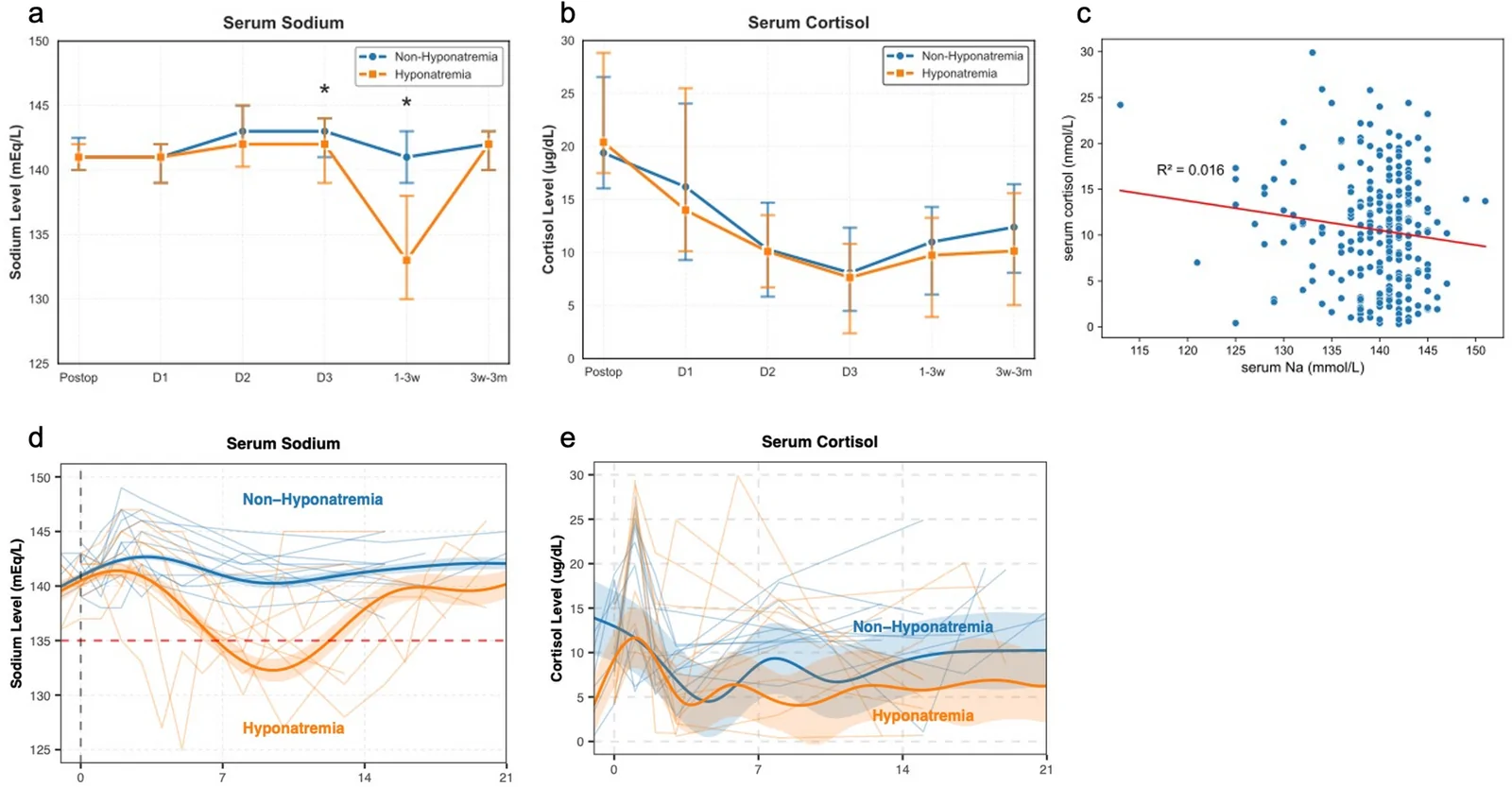
Temporal dynamics of serum sodium and cortisol levels after pituitary surgery. Panel **a**,** b.** Line graphs showing median serum sodium (**a**) and cortisol (**b**) levels at predefined postoperative time points. Error bars represent IQR. The DPH group (orange) and non-DPH group (blue) are compared at immediate postoperative (Postop), days 1–3 (D1-D3), early follow-up (1-3w), and late follow-up (3w-3 m). Asterisks indicate statistically significant differences. Note the divergence in sodium levels at day 3 and early follow-up period, while cortisol levels follow similar declining and recovery patterns in both groups. Panel **c**. Scatter plot showing the relationship between serum sodium and morning cortisol levels during the early outpatient follow-up period. The red regression line and R² value of 0.012 demonstrate the absence of linear correlation between cortisol and sodium levels. Panel **d**,** e**. GAMM analysis illustrating temporal trajectories of serum sodium (**d**) and cortisol (**e**) levels from surgery through 21 days postoperatively. Solid lines represent the fitted smooth functions with 95% confidence intervals (shaded areas). Thin blue and orange lines show individual patient trajectories from 10 randomly selected patients per group (spaghetti plots)

Between the DPH and non-DPH groups, serum sodium levels differed significantly on postoperative day 3 and during early outpatient follow-up (postoperative weeks 1–3) (Fig. 1a, b). The largest between-group difference was observed during the early outpatient period, with median sodium levels of 133 mmol/L in the DPH group versus 141 mmol/L in the non-DPH group. In contrast, serum cortisol levels did not differ between groups during the period of greatest sodium difference. Cortisol levels demonstrated a similar temporal pattern in both groups, with a decline through postoperative day 3 followed by gradual recovery during late follow-up. When plotted against serum sodium during the early outpatient follow-up period, morning cortisol levels showed no linear correlation (R² = 0.016) (Fig. 1c).

GAMM-based analysis further highlighted distinct longitudinal profiles for serum sodium and cortisol following surgery. For serum sodium, a clear group-dependent trajectory was observed: the DPH group exhibited a characteristic U-shaped curve with a nadir during postoperative days 7–14, whereas the non-DPH group maintained stable levels between 140 and 145 mmol/L (Fig. 1d). In contrast, GAMM did not identify a consistent group-level pattern for serum cortisol. Cortisol levels demonstrated an overall trend of rising immediately after surgery, declining through postoperative day 3, and subsequently recovering. Although the DPH group showed a slightly lower trend, the differences were not statistically significant. High inter-patient variability limited the ability of GAMM to model individual cortisol trajectories (Fig. 1e).

Propensity score matching yielded 54 matched pairs from the DPH and non-DPH groups. Post-matching diagnostics confirmed balance across baseline covariates, with all SMDs below 0.1 (Online Resource 2). Within this matched cohort, nadir cortisol levels during the critical postoperative period were compared. No significant difference was found in the median nadir cortisol level between the DPH group (6.10 µg/dL) and the non-DPH group (7.34 µg/dL, *p* = 0.29). Likewise, no significant difference was observed in the proportion of hypocortisolism between the two groups (48.2% vs. 40.7%, *p* = 0.52) (Online Resource 3).

### Risk factors for DPH

In the comparative analysis between the DPH and non-DPH groups, several significant differences were identified across patient, tumor, and surgical characteristics (Table 2). The DPH group had a higher median age (62.5 vs. 55 years, *p* = 0.017). Baseline comorbidities—including hypertension, diabetes mellitus, dyslipidemia, and cardiopulmonary conditions—were similarly distributed between groups. Body composition metrics (total body water, fat mass, and skeletal muscle mass) also showed no significant differences.

**Table 2:** Comparison of baseline characteristics between hyponatremia and non-hyponatremia groups

Variable	Hyponatremia (*n* = 54)	Non-hyponatremia (*n* = 207)	*p*-value
Demographics			
Age	62.5 [51.25–68]	55 [45–65.5]	**0.017**
Sex (male: female)	29: 25	102: 105	0.670
BMI, kg/m²	24.4 [23.0–27.2]	25.5 [23.1–27.8]	0.242
Body composition			
Water proportion, %	51 [47–54]	51 [47–54]	0.741
Extracelluar water proportion, %	20 [18–21]	19 [18–20]	0.638
Skeletal muscle, kg	23.8 [20.7–28.0]	25.2 [21.3–31.1]	0.240
Fat, kg	20.2 [16.0–24.5]	21 [17.2–25.1]	0.345
Comorbidities			
Hypertension	15 (27.8)	65 (31.4)	0.727
Diabetes Melitus	9 (16.7)	22 (10.6)	0.324
Dyslipidemia	16 (29.6)	68 (32.9)	0.774
Cardiac disease	5 (9.3)	12 (5.8)	0.543
Pulmonary disease	1 (1.9)	7 (3.4)	0.891
Hepatic disease	1 (1.9)	3 (1.4)	1
Renal disease	1 (1.9)	6 (2.9)	1
Preoperative endocrinologic deficiencies			
ACTH deficiency	24 (44.4)	61 (29.5)	0.053
Gonadotropin deficiency	35 (64.8)	122 (58.9)	0.529
TSH deficiency	9 (16.7)	18 (8.7)	0.144
GH deficiency	33 (61.1)	126 (60.9)	1
Tumor-related factors			
Pituitary apoplexy	0 (0)	6 (2.9)	0.350
Recurrent tumor	6 (11.1)	26 (12.6)	0.955
Tumor volume (cm^3^)	5.86 [3.54–10.7]	5.54 [3.42–8.63]	0.234
Knosp grade 3–4	20 (37.0)	80 (38.6)	0.830
Pituitary cell lineage			
PIT-1	9 (16.7)	28 (13.5)	0.711
SF-1	29 (53.7)	128 (61.8)	0.352
T-PIT	11 (20.4)	35 (61.9)	0.693
Postoperative outcomes			
Operation time, min	95.5 [74.25–132.5]	84 [64.5–105]	**0.011**
Gross total resection	46 (85.2)	188 (90.8)	0.337
Postoperative pneumocephalus	18 (33.3)	25 (12.1)	**< 0.001**
Postoperative AVP deficiency			
Transient	17 (31.5)	70 (33.8)	0.870
Permanent	5 (9.3)	6 (2.9)	0.091
Postoperative meningitis	4 (7.4)	2 (1.0)	**0.021**

Values are presented as number (%) or median [interquartile range], as appropriate

Boldface indicates statistical significance (*p* < 0.05). *p*-values were calculated using the Mann–Whitney U test for continuous variables and either the chi-squared or Fisher’s exact test for categorical variables, depending on expected cell counts

*ACTH* adrenocorticotropic hormone, *AVP* arginine vasopressin, *BMI* body mass index, *GH* growth hormone, *PIT-1* pituitary transcription factor 1, *SF-1* steroidogenic factor 1, *T-PIT* T-box pituitary transcription factor

Regarding tumor characteristics, tumor volume, recurrence status, and Knosp grade were comparable between groups. Preoperative endocrine profiles showed a trend toward a higher prevalence of ACTH deficiency in the DPH group, although this did not reach statistical significance (44.4% vs. 29.5%, *p* = 0.053). Other hormonal axes, including gonadotropin, TSH, and GH, did not differ significantly between the two groups. Postoperative hydrocortisone replacement during the initial postoperative period was administered in 30 of 54 patients (55.6%) in the DPH group and 96 of 207 patients (46.4%) in the non-DPH group. At the time of hyponatremic episode, 22 of 54 patients (40.7%) were on physiologic hydrocortisone replacement.

Surgical parameters demonstrated distinct group differences. The DPH group had higher rates of postoperative pneumocephalus (33.3% vs. 12.1%, *p* < 0.001) and longer operation times (median 95.5 vs. 84 min, *p* = 0.011). Postoperatively, AVP deficiency occurred in 98 patients (37.5%), of whom 11 (4.2%) were permanent. The incidence of transient AVP deficiency was comparable between the two groups, whereas permanent AVP deficiency was more frequent in the DPH group, although this difference did not reach statistical significance (9.3% vs. 2.9%, *p* = 0.091). Postoperative meningitis occurred significantly more often in the DPH group than in the non-DPH group (7.4% vs. 1.0%, *p* = 0.021).

In univariable analysis, six factors were significantly associated with DPH: age ≥ 60 years, preoperative ACTH deficiency, tumor volume ≥ 10 cm³, longer operation time and postoperative pneumocephalus. Variance inflation factor (VIF) analysis showed no evidence of multicollinearity. Using stepwise backward elimination, the final multivariable logistic regression model included age, postoperative pneumocephalus, preoperative ACTH deficiency, and tumor volume. Among these, age ≥ 60 years (OR 2.09, 95% CI 1.11–3.96, *p* = 0.023) and postoperative pneumocephalus (OR 3.28, 95% CI 1.59–6.81, *p* = 0.001) emerged as independent risk factors for DPH, indicating that the observed age difference between groups persisted after adjustment for confounders (Table 3).

**Table 3:** Univariable and multivariable logistic regression analyses for predictors of delayed postoperative hyponatremia

Parameter	Univariable analysis			Multivariable analysis		
	OR	95% CI	*p*-value	OR	95% CI	*p*-value
Age > 60	2.18	1.18–4.01	**0.012**	2.09	1.11–3.96	**0.023**
Preoperative ACTH deficiency	1.91	1.04–3.54	**0.038**	1.63	0.85–3.15	0.142
Tumor volume$\:\ge\:$10cm^3^	2.38	1.21–4.64	**0.011**	1.92	0.94–3.92	0.075
Operation time (min)	1.01	1.00-1.02	**0.005**			
Postoperative pneumocephalus	3.64	1.80–7.36	**< 0.001**	3.28	1.59–6.81	**0.001**

Boldface indicates statistical significance (*p* < 0.05). *OR* odds ratio, *CI* confidence interval, *ACTH* adrenocorticotropic hormone

### Effects of preoperative ACTH deficiency and perioperative steroid replacement

**Fig. 2:**
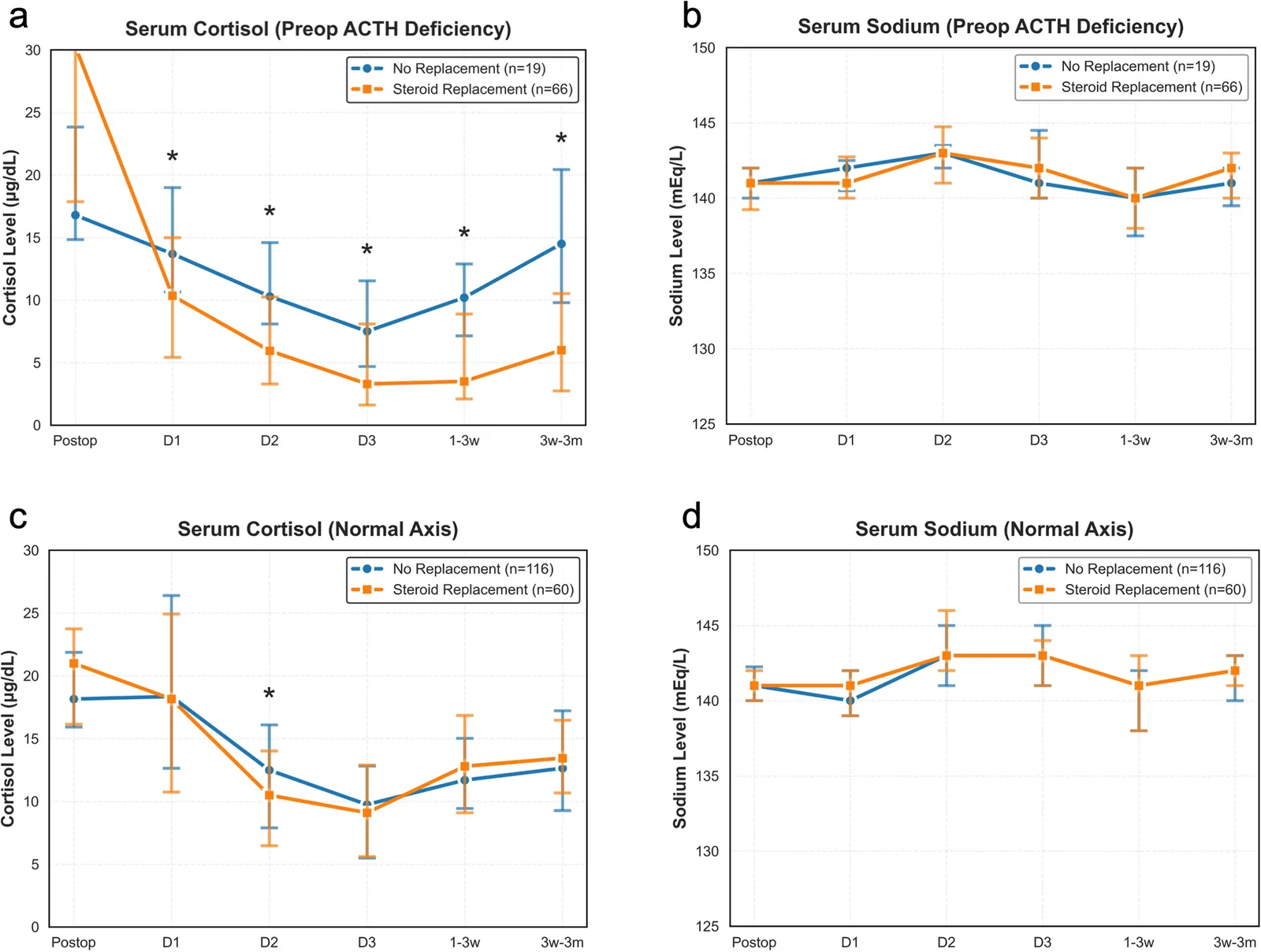
Serial changes in serum sodium and cortisol levels stratified by preoperative ACTH status. Asterisks indicate statistically significant differences. Panel **a**,** b**. Serial cortisol (**a**) and sodium (**b**) levels in patients with preoperative ACTH deficiency, stratified by perioperative hydrocortisone replacement. The steroid replacement group showed significantly lower cortisol levels from postoperative day 2 onward, while sodium levels were comparable between groups. Panel **c**,** d**. Serial cortisol (**c**) and sodium (**d**) levels in patients with a normal ACTH axis. No significant differences were observed between the steroid replacement and no-replacement groups for either cortisol or sodium

As preoperative ACTH deficiency was identified as a factor associated with DPH in univariable analysis, patients were stratified according to HPA axis function to assess whether perioperative steroid replacement could mitigate the risk of DPH. Among the 85 patients with preoperative ACTH deficiency, 24 belonged to the DPH group and 61 to the non-DPH group. Perioperative corticosteroid replacement was administered in the majority of cases (77.6%), with comparable rates between the DPH and non-DPH groups (79.2% vs. 77.0%). In the remaining cases, the rapid ACTH stimulation test was performed on the morning of the operative day, and the results were not available before surgery; in these patients, the decision to initiate hydrocortisone replacement was based on postoperative morning cortisol levels. Patients who received perioperative steroid replacement exhibited significantly lower postoperative serum cortisol levels from postoperative day 2 onward compared with those who did not receive steroid replacement (Fig. 2a, b). Despite this difference in cortisol levels, postoperative serum sodium levels did not differ according to steroid replacement status, and the incidence of DPH was comparable between the replacement and non-replacement subgroups (28.8% vs. 26.3%). Similarly, among patients with a normal preoperative HPA axis, perioperative steroid replacement was not associated with a reduced incidence of DPH, with comparable rates observed between the replacement and non-replacement groups (18.3% vs. 16.4%) (Fig. 2c, d).

## Discussion

DPH represents a significant complication following Pit-NET (also known as pituitary adenoma) surgery, with an incidence of 20.7% in our study. Most cases were identified through routine laboratory monitoring, as symptomatic presentations accounted for about 20%. In our cohort, serum sodium levels reached their nadir at a median of 8 days postoperatively, with over 90% of cases detected within 2 weeks after surgery. This timeline emphasizes the critical importance of monitoring sodium levels during the first two postoperative weeks. Although the majority of DPH cases in this study were asymptomatic at the time of detection, this likely reflects the effectiveness of active postoperative surveillance protocol, in which serum sodium levels were routinely monitored at postoperative weeks 1, 2, and 4. If not detected and managed in a timely manner, severe hyponatremia may result in serious neurological complications such as seizures and altered consciousness. Furthermore, DPH remains the most common cause of unplanned readmission after transsphenoidal surgery, imposing a significant burden on both patients and healthcare systems [20, 21]. These findings underscore the clinical importance of identifying risk factors and understanding the natural course of DPH, even in the absence of overt symptoms at initial detection.

Although symptom severity did not always directly correlate with the degree of hyponatremia, patients with lower sodium levels generally exhibited more pronounced symptoms. Consequently, the severe DPH group demonstrated higher rates of both symptomatic presentation and hospital readmission. For the treatment of hyponatremia, beside fluid restriction applied to all patient postoperatively, corticosteroid replacement therapy was the predominant approach at our institution, with more than half of patients receiving corticosteroids or being managed conservatively with observation and follow-up. This conservative approach proved sufficient for most cases. However, in our experience with severe or symptomatic cases, more aggressive interventions such as readmission and 3% saline infusion were occasionally necessary. Careful patient selection for aggressive therapy is therefore critical. Those with profound sodium deficits and neurological symptoms are the appropriate candidates for intensive intervention, whereas others can often be managed expectantly with safe outpatient follow-up.

An important aspect of our study was the serial tracking of serum cortisol levels from the immediate postoperative period through outpatient follow-up. This longitudinal monitoring of serum sodium and cortisol allowed us to observe the temporal relationship between the two parameters. Additionally, by specifically checking cortisol levels at the time hyponatremia occurred, our study attempted to clarify whether transient adrenal insufficiency precipitates sodium decline. The longitudinal cortisol profiles were broadly similar between the DPH and non-DPH groups. Propensity-score matched analysis also demonstrated no significant correlation. Collectively, these results indicate that hypocortisolism alone is unlikely to be the predominant contributor to DPH in our cohort.

SIADH is regarded as one of the primary causes of delayed postoperative hyponatremia [2, 22, 23]. In contrast, true cerebral salt wasting appears to be relatively rare in this setting [24]. Multiple studies have reported that SIADH is far more commonly implicated than cerebral salt wasting in the pathogenesis of DPH. Since SIADH is characterized by inappropriate AVP secretion, copeptin—a stable C-terminal fragment of the AVP precursor co-secreted in equimolar amounts with AVP—has been investigated as a potential surrogate marker to evaluate the contribution of AVP dysregulation to DPH. In our previous prospective study, preoperative copeptin levels and their ratios to serum sodium and urine osmolarity were significantly lower in patients who subsequently developed DPH [6]. The copeptin-to-serum sodium ratio at baseline yielded the highest predictive performance (AUROC 0.699), but this predictive value was limited to patients without transient AVP deficiency. Binu et al. similarly reported that postoperative copeptin changes could predict sodium disturbances, including delayed hyponatremia [25]. In contrast, Efthymiadis et al. found no evidence supporting copeptin as a predictive marker for post-transsphenoidal SIADH [26]. These conflicting results suggest that DPH is likely a multifactorial phenomenon rather than being driven by SIADH alone, and that the predictive utility of copeptin may depend on the timing of measurement and the presence of concomitant AVP deficiency.

Beyond these mechanistic considerations, our study identified several risk factors that predispose patients to DPH. Advanced age emerged as an important independent predictor. A recent meta-analysis of 27 studies also confirmed age as the only consistent risk factor (pooled OR 1.16) [27]. Araujo-Castro et al. reported that younger age (≤ 65 years) was associated with a higher risk of transient postoperative DI [28], suggesting that age may influence postoperative water-electrolyte disturbances through different mechanisms depending on the type of electrolyte imbalance. One hypothesis is that elderly patients have an impaired ability to handle free water loads due to an age-related decline in renal concentrating capacity or ADH regulation [22]. However, we found no meaningful correlation between any bioimpedance-derived metrics and the development of DPH. This negative finding suggests that the capacity for physiological compensation—such as endocrine and renal responses—is more important than static body composition in determining susceptibility to hyponatremia. Another consideration is that comorbidities and polypharmacy in older patients could contribute to hyponatremia risk, though in our analysis we adjusted for common comorbid conditions.

Another risk factor identified in our analysis was postoperative pneumocephalus. Rather than a direct causative factor, postoperative pneumocephalus serves as an objective surrogate marker for significant intraoperative CSF leakage. Pneumocephalus is readily identifiable on routine postoperative CT, making it a reproducible and easily generalizable variable. Brain shift resulting from pneumocephalus may exert mechanical stress on the pituitary stalk, triggering inappropriate AVP release and subsequent DPH. The association between pneumocephalus and DPH is consistent with prior observations from our institution, where postoperative AVP deficiency correlated with the tumor’s cephalocaudal diameter [29]. This may reflect the influence of vertical tumor extension on pituitary stalk disturbance and, consequently, ADH release. Our findings are also concordant with a previous report demonstrating a higher incidence of postoperative hyponatremia following pituitary transposition, a procedure that involves stalk manipulation [30, 31]. Notably, Knosp grade—an indicator of lateral tumor extension—was not associated with DPH, reinforcing the notion that vertical rather than horizontal tumor growth has greater relevance to hyponatremia risk [32]. While postoperative pneumocephalus is often inevitable due to tumor extent or congenital diaphragmatic defect, our findings underscore the importance of increased clinical awareness for DPH in this subgroup.

The role of hydrocortisone replacement in the management of DPH warrants careful consideration. At our institution, patients who developed postoperative hyponatremia were frequently administered corticosteroids even in the absence of overt adrenal insufficiency, and most of these patients subsequently demonstrated improvement in serum sodium levels. The therapeutic rationale for hydrocortisone replacement in DPH is that hydrocortisone exerts not only glucocorticoid effects but also mineralocorticoid activity, thereby directly promoting sodium reabsorption in the renal tubules [33, 34]. However, because these observations are based on clinical practice, a prospective controlled study is required to determine whether hydrocortisone replacement should be adopted as a standardized component of DPH management.

The preventive role of hydrocortisone in DPH was examined given the higher incidence observed in patients with preoperative ACTH deficiency. Among patients with a normal ACTH axis, corticosteroid replacement was administered only in cases of immediate postoperative hypocortisolism, yet it did not influence the development of DPH. In the ACTH-deficient cohort, those who received steroid replacement exhibited significantly lower cortisol levels from postoperative day 2 onward; nevertheless, this regimen did not reduce the incidence of DPH, suggesting a limited preventive role for preoperative steroids.

There are several limitations to this study. First, it was conducted as a single-center retrospective analysis, which inherently limits data accuracy and restricts the ability to establish causality. Distinguishing SIADH from cerebral salt wasting was challenging due to the lack of systematic data on patients’ volume status, fluid balance, and urinary output during episodes of hyponatremia. As a result, we were unable to assign a definitive etiology for each case, which limits mechanistic interpretation. Second, because most patients were discharged by postoperative day 3 and subsequent follow-up occurred in the outpatient setting, the temporal resolution of both electrolyte and cortisol measurements was suboptimal. This limitation may have led to missed fluctuations in hormone or sodium levels that could have provided additional insight into their dynamic interplay.

## Conclusion

DPH remains a clinically significant complication after pituitary adenoma/Pit-NET surgery. Our findings underscore its multifactorial etiology, identifying advanced age and postoperative pneumocephalus as independent predictors. While hypocortisolism has been hypothesized as a contributing factor, serial cortisol profiling revealed no consistent temporal or quantitative correlation with serum sodium levels, indicating that it is not the primary driver of DPH in most cases. Recognizing high-risk patients may facilitate timely monitoring and intervention. Future prospective studies are essential to validate these observations and to develop effective prophylactic strategies.

## Supplementary Information

Below is the link to the electronic supplementary material.

Supplementary Material 1

## Data Availability

The datasets generated during the current study are available from the corresponding author on reasonable request.
