# Supplementary material for: The Limited Role of Hypocortisolism in the Development of Delayed Postoperative Hyponatremia After Pituitary Surgery
Source: Pituitary. 2026 Jun 16;29(4):100. doi: 10.1007/s11102-026-01702-9 (PMC13272243; doi:10.1007/s11102-026-01702-9)

Online Resources – Pituitary

The Limited Role of Hypocortisolism in the Development of Delayed Postoperative Hyponatremia After Pituitary Surgery

Jong Ha Hwang, MD[1], Hye Seok Park, MD[1], Sun Mo Nam, MD[1], Seung Shin Park, MD, PhD[2], Jung Hee Kim, MD, PhD[2], Min-Sung Kim, MD, PhD[1], Chul-kee Park, MD, PhD[1], Yong Hwy Kim, MD, PhD[1]

[1]Department of Neurosurgery, Seoul National University Hospital, Seoul National University College of Medicine, 101 Daehak-ro, Jongno-gu, Seoul 03080, Republic of Korea

[2]Division of Endocrinology and Metabolism, Department of Internal Medicine, Seoul National University Hospital, Seoul National University College of Medicine, Seoul 03080, Republic of Korea

Corresponding Author: Yong Hwy Kim, MD, PhD

Email: kimyh96@snu.ac.kr

Online Resources 1. (Figure) Characteristics and management of delayed postoperative hyponatremia.

Panel a. Distribution of nadir serum sodium levels and timing in patients with DPH (n=52). Each dot represents an individual patient, with symptomatic cases shown in red and asymptomatic cases in blue. The vertical red line indicates the median time to nadir (8.0 days), and the horizontal blue line represents the median nadir sodium level (129.0 mEq/L). **Panel b.** Treatment modalities and readmission rates stratified by hyponatremia severity. Bar chart showing the proportion of patients receiving each treatment intervention: observation (blue), 3% saline (orange), steroid replacement (green), and readmission (red). The severity categories are defined as mild (130-135 mEq/L), moderate (125-130 mEq/L), and severe (<125 mEq/L).

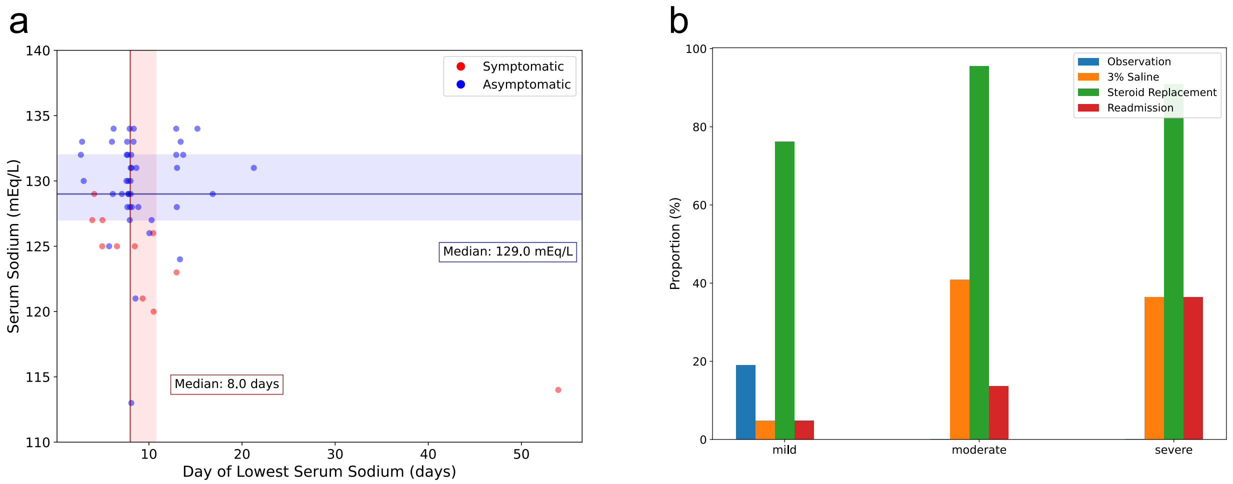

Online Resources 2. (Figure) Love plot displaying standardized mean differences (SMDs) for covariates used in propensity score matching. Red dots represent SMDs before matching, and cyan dots represent SMDs after matching. The vertical dashed line indicates the threshold of 0.1 for adequate balance. Matching was performed using the following variables: age, sex, preoperative ACTH deficiency, operation time, postoperative pneumocephalus, and tumor volume. All covariates achieved SMDs below 0.1 after matching, confirming successful balance between the DPH group and non-DPH group.

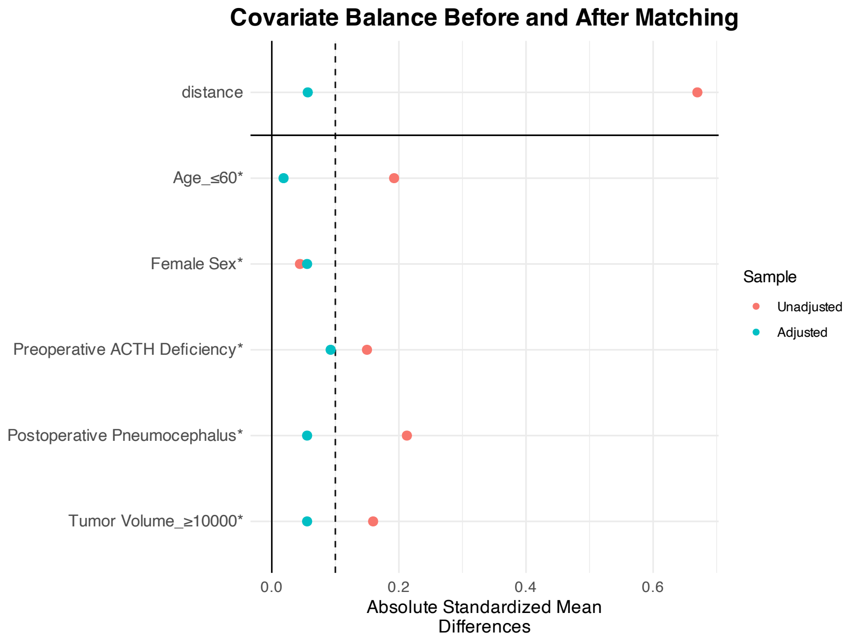

Online Resources 3. (Figure) Propensity score-matched comparison of cortisol levels during hyponatremic episodes. **Panel a.** Box plots comparing nadir cortisol levels between propensity score-matched groups (54 pairs). Each dot represents an individual patient's nadir cortisol during the hyponatremia time window (or corresponding window for controls). The DPH group (green) showed lower median nadir cortisol (6.10 vs 7.34μg/dL, p=0.29). **Panel b.** Comparison of hypocortisolism rates (defined as cortisol <5μg/dL) between matched groups. The DPH group had a higher proportion (OR 1.35), but McNemar's test showed no significant difference (p=0.5235).

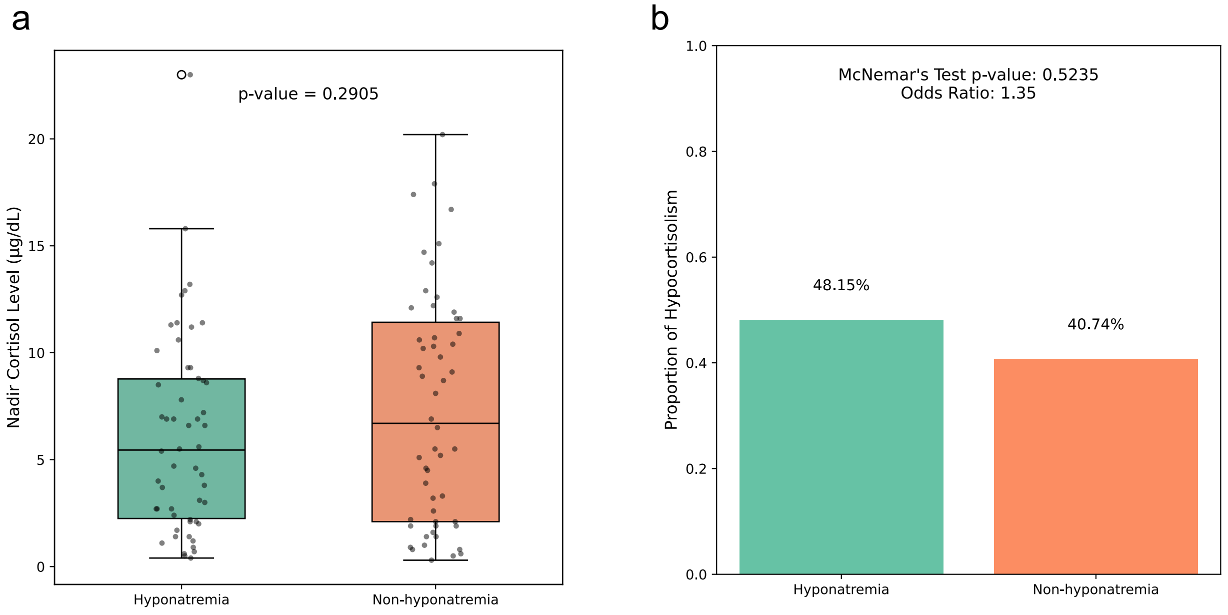

Supplement: Supplementary file 1 — Supplementary Material 1 [file 11102_2026_1702_MOESM1_ESM.pdf]
